# Global, regional, and national trends in the burden of bipolar disorder among women of reproductive age from 1990 to 2021, and projections to 2041: a systematic analysis from the Global Burden of Disease Study 2021

**DOI:** 10.3389/fpsyt.2025.1677304

**Published:** 2025-11-03

**Authors:** Feihu Hu, Huaichen Zhang, Qin He, Jing Zhang, Jingbei Zhang, Chuanfu Song, Cheng Yang

**Affiliations:** ^1^ Department of Psychosomatic Medicine, The Second Affiliated Hospital of Nanchang University, Nanchang, China; ^2^ Department of Psychiatry, Daizhuang Hospital, Jining, Shandong, China; ^3^ Department of Psychiatry, The Fourth People’s Hospital of Wuhu, Wuhu, Anhui, China

**Keywords:** bipolar disorder, women of reproductive age, Global Burden of Disease, disability-adjusted life years, sociodemographic index

## Abstract

**Background:**

Bipolar disorder (BD) is a severe mental illness characterized by alternating episodes of mania and depression. Among women of reproductive age (15–49 years), the risk of onset is higher and clinical manifestations are more complex due to the combined influence of hormonal fluctuations, reproductive pressures, and conflicts in social roles. Mental health problems in this population not only significantly impair quality of life but also place a sustained burden on family stability and public health systems. Although awareness of BD has been increasing, studies specifically focusing on the disease burden among women of reproductive age remain limited, particularly lacking systematic analyses based on the latest Global Burden of Disease (GBD) data.

**Methods:**

This study was based on data from the GBD 2021 and systematically analyzed the incidence, prevalence, and disability-adjusted life years (DALYs) of BD among women aged 15–49 at the global, regional, and national levels from 1990 to 2021. We calculated age-standardized incidence rate (ASIR), prevalence rate (ASPR), and DALYs rate (ASDR), along with the estimated annual percentage change (EAPC) for each metric. In addition, cross-national health inequality was assessed using the slope index of inequality (SII) and the concentration index (CII). Future trends in the disease burden were projected to 2041 using a Bayesian age–period–cohort (BAPC) model.

**Results:**

From 1990 to 2021, the overall burden of BD among women aged 15–49 years has continued to rise globally. In 2021, the numbers of new cases, prevalent cases, and DALYs were all significantly higher than in 1990. Although the ASIR showed a slight decline (EAPC = −0.07; 95% CI: −0.08 to −0.05), the ASPR and ASDR continued to increase, with EAPC of 0.06 (95% CI: 0.04–0.07) and 0.05 (95% CI: 0.04–0.07), respectively. High-SDI regions ranked highest across all three metrics, while middle-high SDI regions had the lowest ASIR and ASPR, and middle-low SDI regions had the lowest ASDR. Regionally, South Asia bore the heaviest absolute burden in terms of incident cases, prevalence, and DALYs, whereas Oceania had the lightest burden. In terms of standardized rates, Tropical Latin America had the highest ASIR, while Australasia recorded the highest ASPR and ASDR; East Asia had the lowest values across all three indicators. At the national level in 2021, New Zealand reported the highest ASIR, ASPR, and ASDR globally; China had the lowest ASIR, and the Democratic People’s Republic of Korea had the lowest ASPR and ASDR. The burden of BD was positively correlated with the level of socio-demographic development, showing a mild positive correlation between ASIR and SDI, and a stronger correlation for ASPR and ASDR. Age-wise, all age groups saw increases in incident cases, prevalence, and DALYs compared to 1990, with the 35–39 age group showing the greatest rise in incidence, and the 45–49 group the largest increase in prevalence and DALYs. In 2021, the ASIR peaked in the 15–19 age group, while ASPR and ASDR were highest in the 25–29 age group. Health inequality analysis indicated persistent disparities in BD burden between high- and low-SDI countries, with little improvement over the past three decades. Projections using the BAPC model suggest that ASIR, ASPR, and ASDR may slightly decline by 2041, but the overall disease burden is expected to remain high.

**Conclusion:**

From 1990 to 2021, the disease burden of BD among women of reproductive age has continued to rise globally, exhibiting significant regional, national, and socioeconomic disparities. This highlights the uneven development of mental health systems across different settings. Countries should develop more targeted mental health intervention strategies based on their stage of development. It is recommended to strengthen early screening, standardized diagnosis, and long-term comprehensive management for women of reproductive age, in order to effectively reduce the disease burden and improve overall mental health in this population.

## Introduction

Bipolar disorder (BD) is a severe mental illness characterized by extreme mood fluctuations, with alternating episodes of mania and depression (1). It is often accompanied by impaired attention, cognitive dysfunction, and reduced social adaptability (2). BD is marked by high recurrence and chronicity, leading to a substantial disability rate and significantly affecting patients’ daily lives and social functioning (3). Epidemiological studies have consistently demonstrated a stable sex disparity in BD worldwide, with women exhibiting higher incidence and disability burdens than men (4). Women of reproductive age (15–49 years) are particularly vulnerable, as hormonal fluctuations combined with the intersecting pressures of reproduction, employment, and family caregiving substantially increase their risk of developing BD (5–7). Mental health issues in this population not only compromise individual well-being but may also impact reproductive health, parenting capacity, family stability, and overall labor force availability. Therefore, a comprehensive understanding of the epidemiological patterns and burden of BD among women of reproductive age is critical for strengthening mental health service systems tailored to women, promoting early identification and targeted interventions, and advancing global efforts toward gender-equitable health outcomes.

Although research on the burden of BD has increased in recent years, most studies have focused on the general population (8) or specific geographic regions (9), with limited analyses specifically targeting women of reproductive age. Existing literature is primarily based on data from the 2019 (10) or earlier iterations of the Global Burden of Disease (GBD) study (11) and does not fully capture the potential impact of the COVID-19 pandemic on healthcare resource allocation, increased pressure on mental health service systems, or shifts in social structures and cultural norms that may profoundly affect the mental health of this population (12). Moreover, substantial cross-national differences in diagnostic criteria, accessibility of psychiatric care, public awareness, and gender-related biases may introduce estimation bias or lead to underestimation of the true disease burden. Critically, the lack of systematic comparative research based on the Sociodemographic Index (SDI) limits the ability to formulate evidence-based, targeted interventions and optimize resource allocation for high-risk female populations at global and regional levels.

In light of the current research gaps, this study utilized data from the GBD 2021 (13) to systematically analyze the epidemiological characteristics and evolving burden of BD among women aged 15–49 at global, regional, and national levels from 1990 to 2021. The analysis focused on key metrics including incidence, prevalence, and disability-adjusted life years (DALYs), along with their corresponding age-standardized rates. The estimated annual percentage change (EAPC) was used to quantify long-term temporal trends. Additionally, from a health inequality perspective, the study examined disparities in BD burden across countries with different levels of sociodemographic development. Furthermore, a Bayesian age–period–cohort (BAPC) model was constructed to project trends in BD burden through 2041. This study aims to provide data-driven support for optimizing women’s mental health service systems, inform stratified intervention strategies, and offer theoretical and policy guidance for addressing the growing public health challenge of mental disorders among women of reproductive age worldwide.

## Methods

### Data source

This study is based on the GBD 2021 database and systematically extracted the incidence (annual new cases), prevalence (total existing cases), and DALYs (years of life lost due to premature death and disability) of BD among women aged 15–49 across 204 countries and territories, along with the corresponding age-standardized rates, to comprehensively reflect the disease burden in this population. The GBD data are derived from multiple sources, including vital registration, epidemiological surveys, population censuses, household health surveys, and other monitoring channels, and have been standardized and cross-regionally calibrated to ensure the scientific rigor, representativeness, and comparability of the estimates. This study represents a secondary analysis of the GBD data, fully leveraging its high-quality and globally consistent resources to ensure the reliability of the findings. The results provide evidence-based support for the development of mental health interventions and public health policies targeting women of reproductive age, and inform the optimal allocation of global mental health resources.

### Disease definition

In the GBD 2021 disease classification framework, bipolar disorder is categorized as a level 3 diagnosis under the broader level 2 category of mental disorders and the level 1 category of non-communicable diseases (14). As a major subtype of mood disorders, bipolar disorder is characterized by alternating episodes of mania and depression, often accompanied by significant mood instability, cognitive impairment, and social dysfunction. For disease coding purposes, bipolar disorder is clearly defined within the International Classification of Diseases (ICD), falling under the range of F31.0 to F31.9 in ICD-10 (15). This standardized classification provides a consistent framework for global burden estimation, epidemiological research, and clinical management.

### Age groups

To better characterize the age-specific distribution of bipolar disorder among women of reproductive age, the population aged 15–49 was stratified into seven consecutive age groups: 15–19, 20–24, 25–29, 30–34, 35–39, 40–44, and 45–49 years. This grouping approach accounts for the distinct physiological and reproductive stages women experience—including adolescence, peak fertility, and perimenopause—and aims to reveal dynamic patterns of disease burden across the life course. Such stratification provides a scientific basis for age-specific prevention, targeted interventions, and service optimization.

### SDI

SDI is a composite metric developed within the GBD framework to capture the relationship between a location’s level of socioeconomic development and its health outcomes. It is calculated as the geometric mean of three key normalized indicators: the total fertility rate among individuals under age 25, the mean years of education for those aged 15 and older, and lag-distributed income per capita. SDI values range from 0 to 1, where 0 represents the lowest level of development relevant to health, and 1 indicates the highest. Based on the GBD 2021 classification, countries and territories are grouped into five SDI levels: low (<0.47), low-middle (0.47–0.62), middle (0.62–0.71), high-middle (0.71–0.81), and high (>0.81) (16). SDI serves as a critical stratification tool in burden of disease research, enabling the assessment of socioeconomic disparities in health and guiding the design of context-appropriate intervention strategies and resource allocation at global, regional, and national levels.

### Cross-country inequality analysis

Continuous monitoring of health inequality is essential for implementing precise public health interventions and optimizing the allocation of health resources. In this study, we used the Slope Index of Inequality (SII) and the Concentration Index (CII) to systematically assess disparities in the burden of BD among women of reproductive age across countries with different levels of socioeconomic development (17). The SII is formally defined as the regression slope obtained by ranking countries according to their SDI from lowest to highest and performing a weighted linear regression of the cumulative population proportion against the cumulative burden of disease. It quantifies the absolute difference in disease burden across the socioeconomic gradient, with larger values indicating greater inequality. The CII is defined based on the Lorenz curve, measuring the relative concentration of disease burden across the SDI distribution. Its values range from -1 to 1, where positive values indicate a concentration of burden in higher SDI countries, and negative values indicate concentration in lower SDI countries. These complementary indicators provide both absolute and relative perspectives on the global distribution of BD burden, offering quantitative evidence to guide stratified intervention strategies, optimize resource allocation, and promote equity in mental health.

### Predictive analysis

To predict the future global burden of BD among women of reproductive age, we employed the BAPC model. This approach integrates the effects of age, period, and birth cohort into a Bayesian inference framework to estimate parameters and dynamically simulate long-term disease trends in specific populations. Compared with traditional forecasting methods, the BAPC model provides higher fitting accuracy and predictive performance, particularly for psychiatric disorders characterized by complex temporal structures and evident periodic patterns, and has been widely applied in epidemiological trend analyses. The model was implemented in R software (version 4.4.2) using the “INLA” and “BAPC” packages. We calculated 95% uncertainty intervals (UI) to capture the expected range of uncertainty. Although external validation using independent out-of-sample global datasets was limited due to data availability, the predicted trends were interpreted in the context of historical observations from 1990 to 2021. The predictive results of this study provide evidence-based support for early intervention and offer valuable insights for the optimization of mental health management strategies among women of reproductive age worldwide (18, 19).

### Statistical analyses

In this study, we conducted a systematic assessment and multidimensional analysis of the burden of BD among women of reproductive age (15–49 years) worldwide, using R software (version 4.4.2) and the JD_GBDR platform (version 2.37) developed by Jingding Medical Technology Co., Ltd. To elucidate the influence of socioeconomic factors on the distribution of disease burden, the “health disparities” package in R was used to calculate the SII and the CII. To identify temporal patterns in disease burden, LOESS smoothing was employed for trend visualization, and Spearman rank correlation analysis was performed to assess statistical associations with the SDI. Age-standardized rates were calculated based on the global standard population structure provided by GBD 2021, and a log-linear regression model was applied to compute the EAPC and its 95% confidence interval (CI), quantifying the magnitude of temporal trends. For burden forecasting, we constructed and applied the BAPC model, integrating historical data with age, period, and cohort effects to project future trends in the prevalence of BD. To enhance the robustness and credibility of the estimates, we performed 1,000 posterior distribution samplings to derive the 95% UI for all indicators. All statistical inferences were based on two-sided tests with a significance level of α = 0.05, ensuring the scientific rigor and reliability of the study findings.

## Results

### Global trends

**Figure 1:**
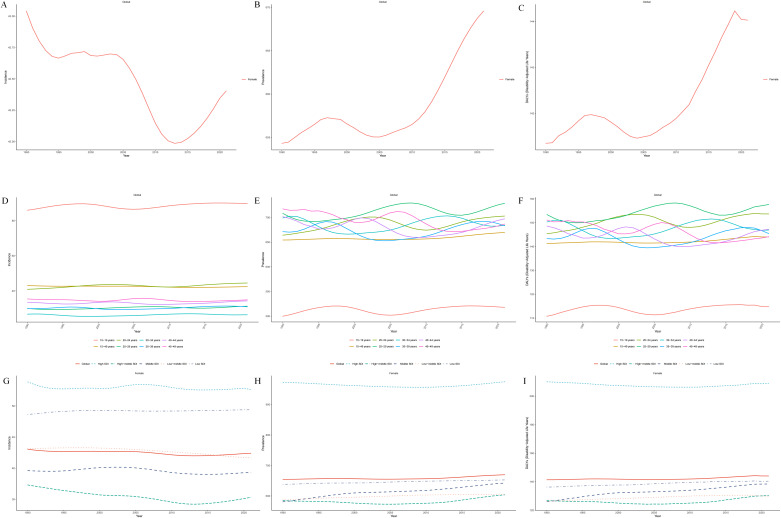
Global trends in the ASIR **(A)**, ASPR **(B)**, and ASDR **(C)** of BD among women of reproductive age (15–49 years) from 1990 to 2021;Trends in ASIR **(D)**, ASPR **(E)**, and ASDR **(F)** of BD among women of reproductive age by age group from 1990 to 2021;Trends in ASIR **(G)**, ASPR **(H)**, and ASDR **(I)** of BD among women of reproductive age across five SDI quintiles from 1990 to 2021.

In 2021, an estimated 826,396.70 (95% UI: 657,695.90–1,051,382.87) new cases of BD occurred among women of reproductive age (15–49 years) worldwide, marking a 43.6% increase from 575,624.58 cases (95% UI: 459,342.10–728,960.22) in 1990. Despite the continuous rise in incident cases, the ASIR showed a slight decline, decreasing from 43.04 per 100,000 population (95% UI: 34.35–54.51) in 1990 to 42.40 per 100,000 (95% UI: 33.75–53.95) in 2021, with an EAPC of −0.07 (95% CI: −0.08 to −0.05) (**Table 1**; **Figure 1A**). Meanwhile, the number of prevalent cases increased from 8,750,596.06 (95% UI: 6,983,783.18–10,912,233.64) in 1990 to 13,049,344.52 (95% UI: 10,393,773.49–16,253,372.49) in 2021. The ASPR also rose from 654.32 per 100,000 (95% UI: 522.21–815.95) to 669.59 per 100,000 (95% UI: 533.33–834.00), with an EAPC of 0.06 (95% CI: 0.04–0.07) (**Table 1**; **Figure 1B**). During the same period, the ASDR increased from 141.35 per 100,000 (95% UI: 92.82–208.03) to 144.03 per 100,000 (95% UI: 93.51–210.62), with an EAPC of 0.05 (95% CI: 0.04–0.07). Furthermore, the total number of DALYs in this population reached 2,806,894.15 (95% UI: 1,822,323.33–4,104,600.83) in 2021, significantly higher than the 1,890,347.28 (95% UI: 1,241,353.75–2,782,076.95) reported in 1990 (**Table 1**; **Figure 1C**).

**Table 1:** Global incidence, prevalence, DALYs, age-standardized rates, and EAPC of BD among women of reproductive age (15–49 years) from 1990 to 2021.

Location	Age group	Measure	1990		2021		1990-2021
Global			All-ages cases	Age-standardized rates per 100,000 people	All-ages cases	Age-standardized rates per 100,000 people	EAPC
			n (95%CI)	n (95% CI)	n (95%CI)	n (95% CI)	n (95% CI)
	15-49years	DALYs	1890347.28(1241353.75,2782076.95)	141.35(92.82,208.03)	2806894.15(1822323.33,4104600.83)	144.03(93.51,210.62)	0.05(0.04,0.07)
		Prevalence	8750596.06(6983783.18,10912233.64)	654.32(522.21,815.95)	13049344.52(10393773.49,16253372.49)	669.59(533.33,834.00)	0.06(0.04,0.07)
		Incidence	575624.58(459342.10,728960.22)	43.04(34.35,54.51)	826396.70(657695.90,1051382.87)	42.40(33.75,53.95)	-0.07(-0.08,-0.05)
	15-19years	DALYs	282985.72(170044.17,443447.58)	110.74(66.54,173.53)	348396.68(207475.80,550317.68)	114.74(68.33,181.23)	0.07(0.02,0.12)
		Prevalence	1276792.14(925137.71,1760159.66)	499.65(362.03,688.80)	1572130.32(1132740.14,2198185.90)	517.74(373.04,723.92)	0.06(0.02,0.11)
		Incidence	219708.76(160448.11,295871.72)	85.98(62.79,115.78)	272589.37(197745.99,370135.03)	89.77(65.12,121.90)	0.09(0.04,0.14)
	20-24years	DALYs	354851.94(224485.16,544316.72)	145.35(91.95,222.96)	451478.16(282364.28,686846.26)	153.69(96.12,233.82)	0.11(0.06,0.17)
		Prevalence	1621142.17(1193240.57,2186191.19)	664.04(488.76,895.48)	2064800.23(1504243.37,2824860.42)	702.91(512.08,961.65)	0.11(0.05,0.16)
		Incidence	99853.79(47460.72,171107.90)	40.90(19.44,70.09)	130704.51(62044.97,224816.54)	44.49(21.12,76.53)	0.18(0.12,0.24)
	25-29years	DALYs	337762.76(213723.95,508174.93)	153.46(97.10,230.88)	458783.96(286113.34,689393.51)	157.66(98.33,236.92)	0.13(0.07,0.18)
		Prevalence	1559302.11(1167996.56,2096245.88)	708.45(530.67,952.41)	2120706.35(1568898.74,2877783.26)	728.80(539.16,988.97)	0.12(0.07,0.17)
		Incidence	66047.80(35182.54,105683.60)	30.01(15.98,48.02)	91015.13(48386.77,146159.19)	31.28(16.63,50.23)	0.16(0.11,0.20)
	30-34years	DALYs	285702.50(182455.08,424617.71)	150.28(95.97,223.35)	439201.32(277040.44,652916.33)	146.92(92.68,218.42)	0.04(-0.03,0.11)
		Prevalence	1328779.50(1010185.14,1728054.18)	698.96(531.37,908.98)	2046018.50(1543547.34,2682684.83)	684.45(516.36,897.43)	0.04(-0.04,0.11)
		Incidence	50832.09(27248.47,85793.35)	26.74(14.33,45.13)	78835.58(42029.77,134243.35)	26.37(14.06,44.91)	0.07(-0.01,0.14)
	35-39years	DALYs	248644.50(156428.53,369143.25)	143.35(90.18,212.82)	404016.30(256093.08,601076.59)	145.43(92.19,216.37)	0.04(-0.03,0.12)
		Prevalence	1164217.54(899074.63,1485842.14)	671.20(518.34,856.62)	1897532.47(1451784.55,2436192.25)	683.05(522.60,876.95)	0.04(-0.04,0.12)
		Incidence	51890.33(25509.84,82709.80)	29.92(14.71,47.68)	85927.15(41131.91,138919.43)	30.93(14.81,50.01)	0.12(0.05,0.19)
	40-44years	DALYs	208431.19(129791.06,302511.43)	148.64(92.56,215.73)	365351.04(226040.29,534840.61)	147.27(91.11,215.58)	-0.07(-0.14,0.00)
		Prevalence	983898.26(767443.41,1241225.88)	701.65(547.29,885.16)	1729864.14(1332002.20,2195864.29)	697.27(536.90,885.11)	-0.07(-0.15,0.00)
		Incidence	47010.57(23319.76,76699.87)	33.52(16.63,54.70)	85084.14(42082.93,139298.73)	34.30(16.96,56.15)	0.04(-0.02,0.11)
	45-49years	DALYs	171968.67(104318.81,257894.40)	151.11(91.67,226.62)	339666.69(205001.58,510659.78)	144.14(87.00,216.71)	-0.20(-0.25,-0.15)
		Prevalence	816464.34(627318.51,1036548.67)	717.45(551.24,910.85)	1618292.51(1234776.42,2072645.63)	686.76(524.00,879.57)	-0.20(-0.25,-0.15)
		Incidence	40281.24(24912.16,57168.92)	35.40(21.89,50.24)	82240.83(50985.20,116870.43)	34.90(21.64,49.60)	-0.07(-0.12,-0.01)

DALYs, disability-adjusted life years; UI, uncertainty interval; CI, confidence Interval; EAPC, estimated annual percentage change

### Global trends by age groups

Between 1990 and 2021, the number of new cases, prevalent cases, and DALYs related to BD increased across all age groups among women aged 15–49 globally. The most pronounced growth in incidence was observed in the 35–39 age group, reaching 85,927.15 cases in 2021—a 65.6% increase from 1990. In contrast, the largest increases in prevalence and DALYs were recorded in the 45–49 age group, with 1,618,292.51 prevalent cases and 339,666.69 DALYs in 2021, representing increases of 98.3% and 97.5%, respectively, compared with 1990 (**Table 1**). In terms of the ASIR, most age groups showed an upward trend, except for slight declines in the 30–34 and 45–49 age groups. Adolescent females aged 15–19 consistently had the highest ASIR across all groups, increasing from 85.98 per 100,000 (95% UI: 62.79–115.78) in 1990 to 89.77 per 100,000 (95% UI: 65.12–121.90) in 2021. The ASIR trend for this group followed a U-shaped pattern—declining gradually from 1990 to 2005, followed by an upward rebound thereafter (**Table 1**; **Figure 1D**). For ASPR and ASDR, all age groups experienced varying degrees of increase, except for slight declines in the 40–44 and 45–49 age groups. The most marked rise was observed in the 20–24 age group (**Table 1**; **Figures 1E, F**). By 2021, the 15–19 age group had the highest ASIR globally, while the 25–29 age group recorded the highest ASPR and ASDR, highlighting the substantial disease burden in this age range (**Table 1**).

### SDI regional trends

From 1990 to 2021, the number of new cases, prevalent cases, and DALYs associated with BD among women aged 15–49 increased across all SDI regions globally. The most substantial increases were observed in low-SDI regions, followed by low-middle SDI regions (**Supplementary Table 1**). Regarding ASIR, only low-SDI regions exhibited an upward trend, while all other SDI categories showed varying degrees of decline, with the most pronounced decrease seen in high-middle SDI regions, followed by low-middle SDI regions. In contrast, ASPR and ASDR showed increasing trends in all SDI regions except high-SDI areas. The most notable increases were found in middle and low-middle SDI regions (**Figures 1G–I**). Notably, by 2021, high-SDI regions had the highest ASIR, ASPR, and ASDR among all SDI categories, whereas high-middle SDI regions had the lowest ASIR and ASPR, and low-middle SDI regions had the lowest ASDR (**Supplementary Table 1**). These findings highlight marked disparities in the burden of BD across different stages of socioeconomic development and reflect persistent global challenges in public health resource allocation and mental health service delivery for women of reproductive age.

### Regional trends

**Figure 2:**
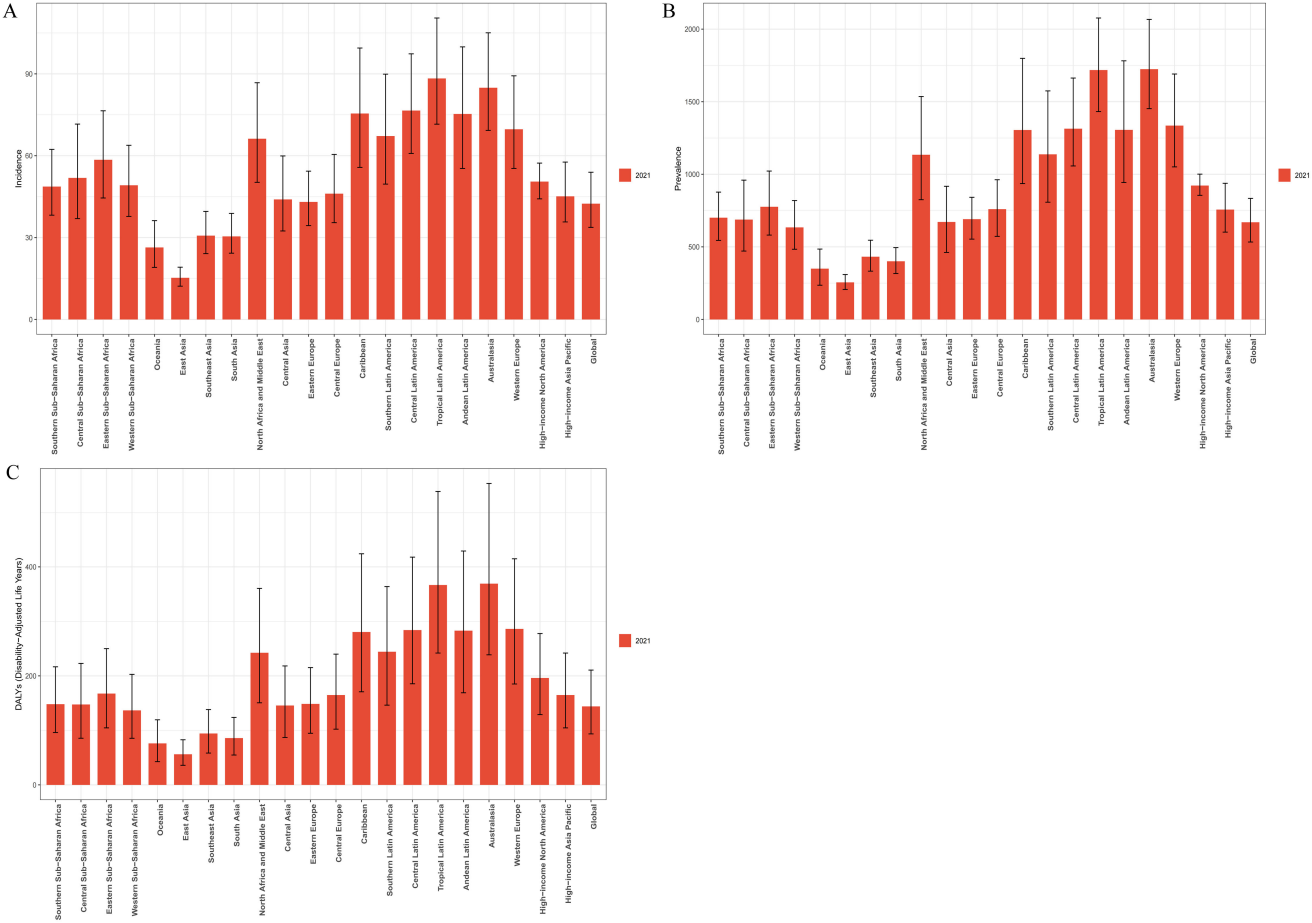
Regional variations in the ASIR **(A)**, ASPR **(B)**, and ASDR **(C)** of BD among women of reproductive age (15–49 years) across 21 global regions in 2021.

**Figure 3:**
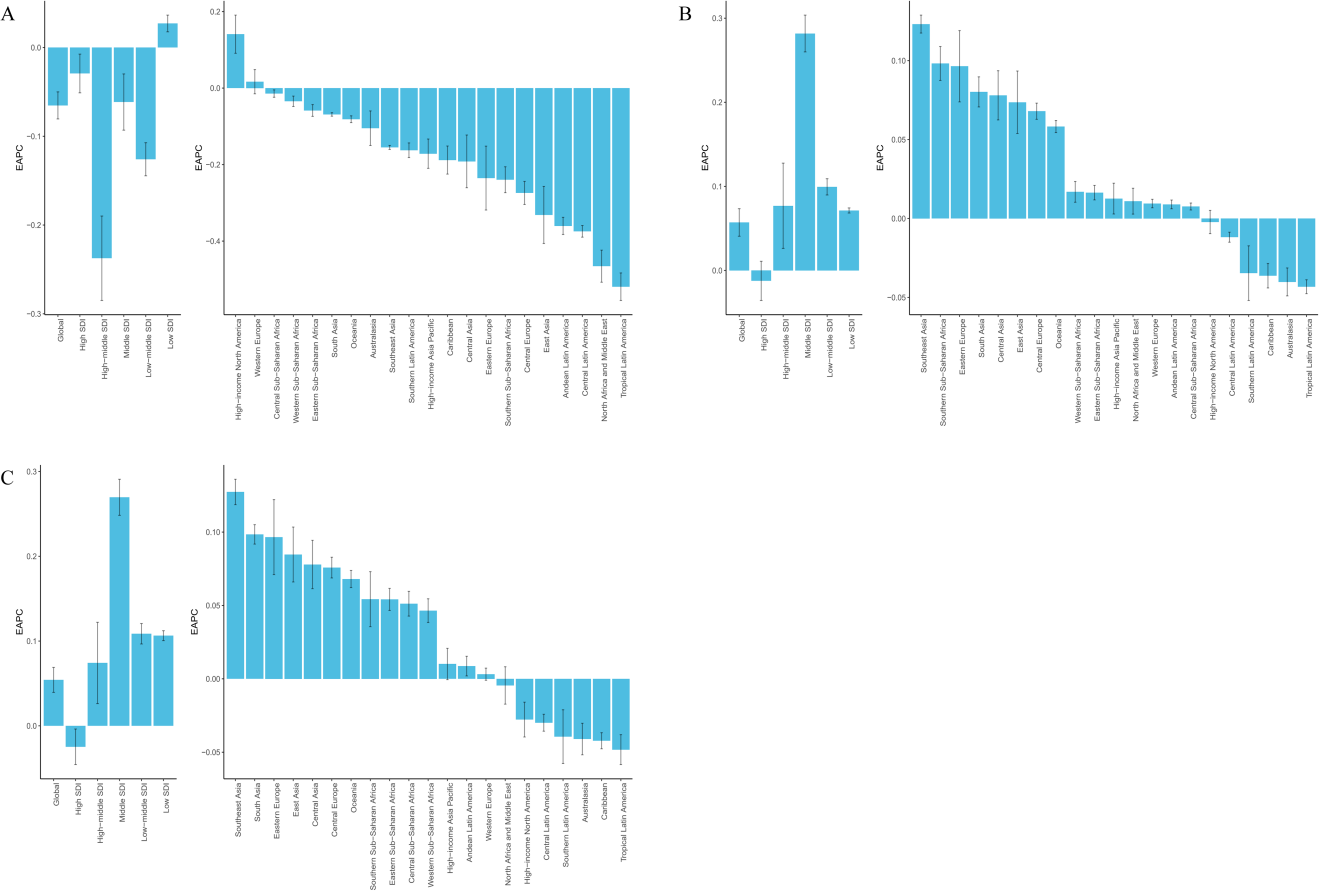
EAPC in age-standardized rates of BD among women of reproductive age (15–49 years) across 21 GBD regions and 5 SDI quintiles from 1990 to 2021.**(A)**. EAPC of ASIR; **(B)** EAPC of ASPR; **(C)** EAPC of ASDR.

**Figure 4:**
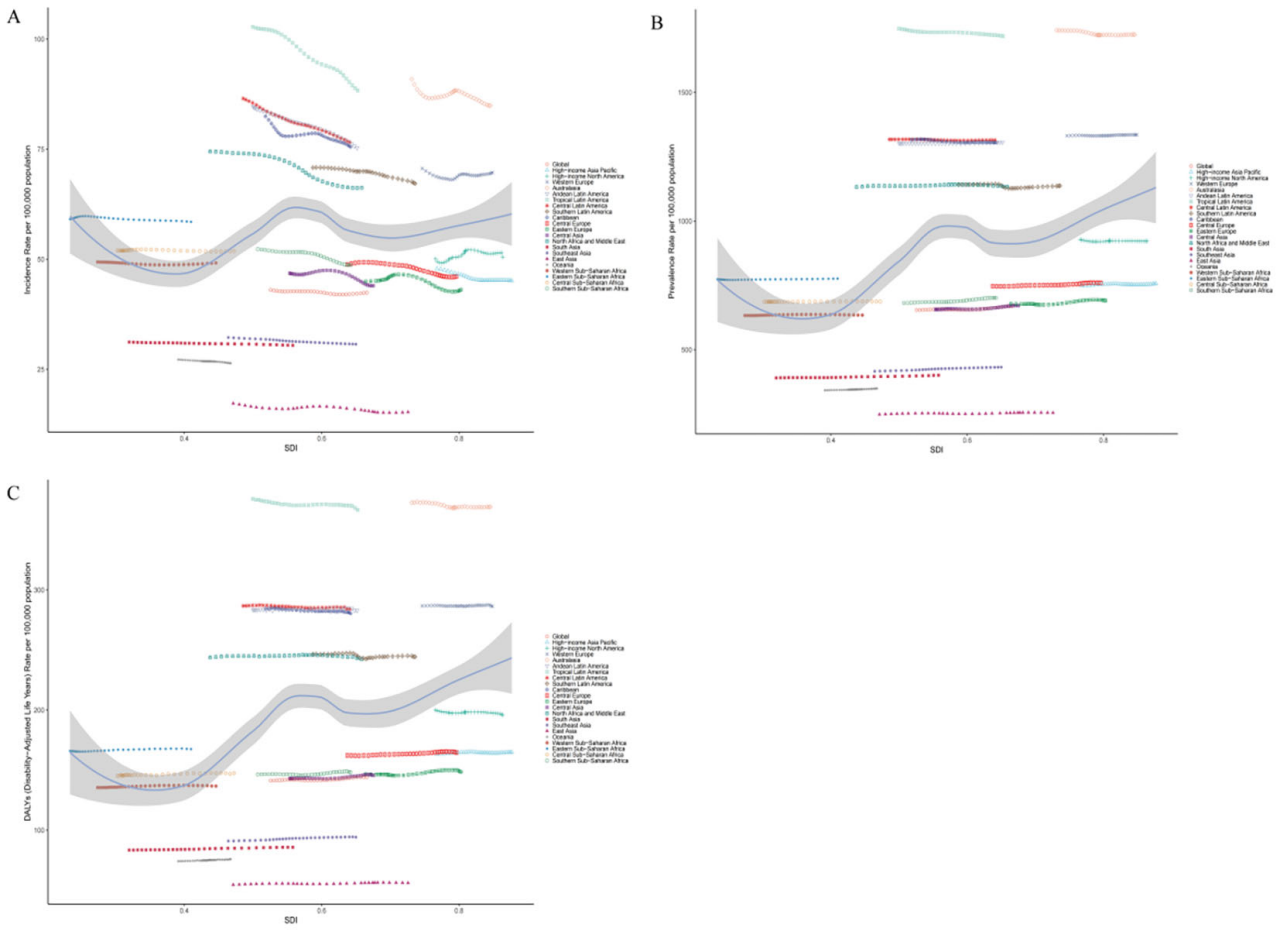
Associations between the SDI and the burden of BD among women of reproductive age across the 21 GBD regions: **(A)**. Association between the ASIR of BD and SDI;**(B)**. Association between the ASPR of BD and SDI;**(C)**. Association between the ASDR of BD and SDI.

At the regional level, as of 2021, the burden of BD among women aged 15–49 varied markedly across global regions. South Asia ranked highest in terms of absolute burden, including new cases, prevalent cases, and DALYs, with 150,377.43 incident cases (95% UI: 119,961.25–191,994.93), 1,980,513.56 prevalent cases (95% UI: 1,563,644.28–2,444,911.80), and 423,922.17 DALYs (95% UI: 270,528.60–611,412.98). Oceania reported the lowest burden, with only 915.27 new cases (95% UI: 662.26–1,256.57), 12,129.73 prevalent cases (95% UI: 8,177.50–16,827.20), and 2,637.21 DALYs (95% UI: 1,478.86–4,144.35) (**Supplementary Table 1**). Significant variation was also observed in age-standardized rates. Tropical Latin America had the highest ASIR at 88.34 per 100,000 (95% UI: 71.56–110.47), while Australasia ranked highest in ASPR and ASDR, at 1,724.25 per 100,000 (95% UI: 1,452.31–2,067.16) and 369.21 per 100,000 (95% UI: 238.64–553.10), respectively. By contrast, East Asia had the lowest values across all three metrics: ASIR of 15.27 per 100,000 (95% UI: 12.19–19.15), ASPR of 255.18 per 100,000 (95% UI: 206.31–309.08), and ASDR of 56.14 per 100,000 (95% UI: 35.90–82.74) (**Supplementary Table 1**; **Figures 2A–C**). Trend analysis revealed a general decline in ASIR across most regions since 1990, with the exception of High-income North America and Western Europe, where increases were observed. The steepest ASIR decline was found in Tropical Latin America (EAPC = –0.52; 95% CI:–0.56 to –0.48). ASPR increased in 15 regions, with the largest rise in Southeast Asia (EAPC = 0.12; 95% CI: 0.12–0.13), while 6 regions saw a decline, most notably Tropical Latin America (EAPC = –0.04; 95% CI: –0.05 to –0.04). ASDR rose in 14 regions, again with Southeast Asia experiencing the largest increase (EAPC = 0.13; 95% CI: 0.12–0.14), whereas declines were observed in 7 regions, with the most significant decrease in Tropical Latin America (EAPC = –0.05; 95% CI:–0.06 to–0.04) (**Supplementary Table 1**; **Figures 3A–C**). Regarding the association between SDI and disease burden, from 1990 to 2021, a weak but statistically significant positive correlation was observed between ASIR and SDI across the 21 GBD regions (Spearman’s r = 0.09; 95% CI: 0.02–0.15; p = 2.3×10⁻²), suggesting slightly higher incidence rates in more developed regions. In comparison, stronger positive correlations were found between SDI and both ASPR (r = 0.37; 95% CI: 0.32–0.43; p < 0.01) and ASDR (r = 0.38; 95% CI: 0.32–0.44; p < 0.01), indicating that as socioeconomic development increases, the prevalence and disability burden of BD among women of reproductive age tend to rise significantly (**Figures 4A–C**).

### National trends

**Figure 5:**
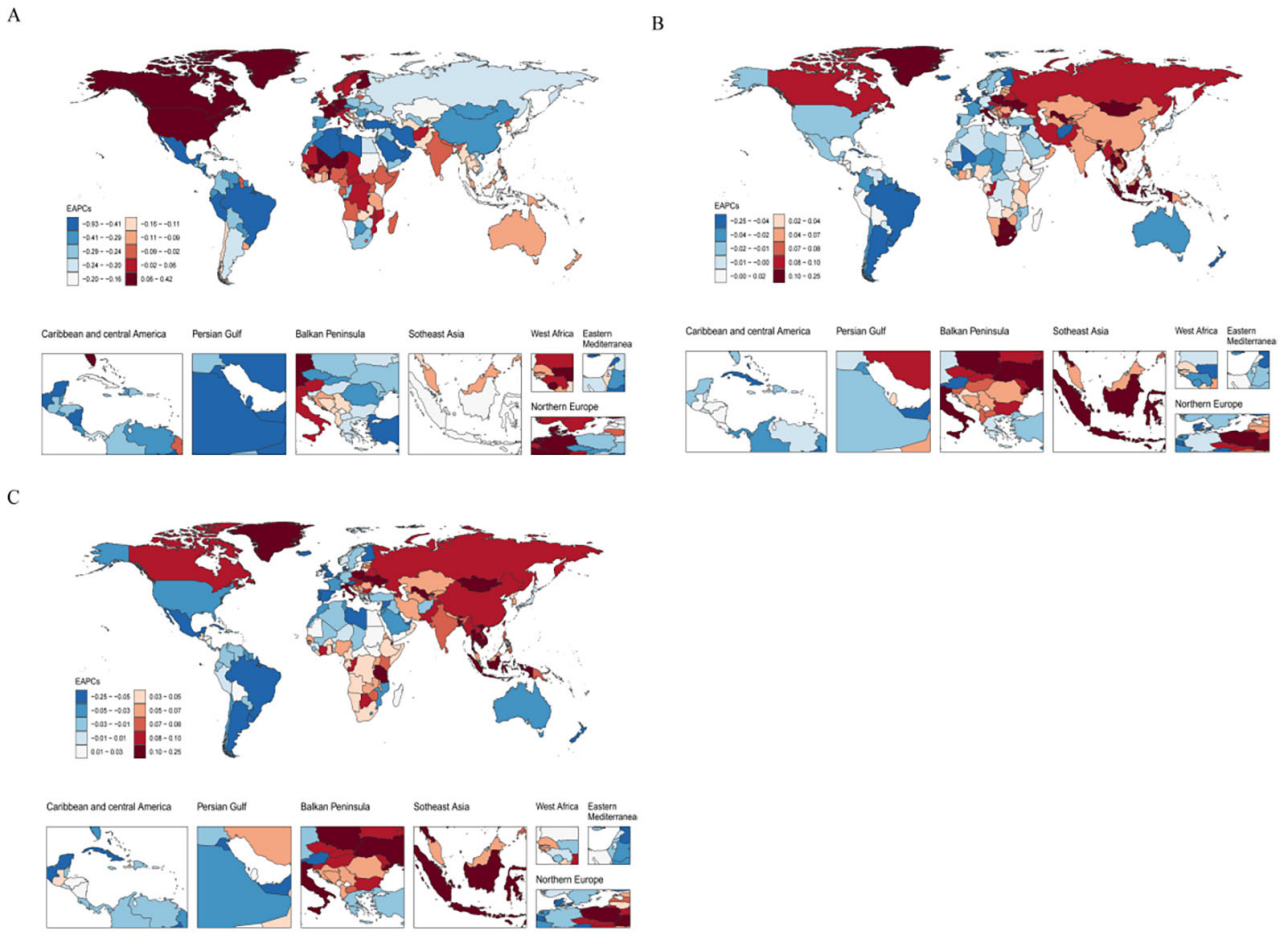
Distribution of the EAPC in age-standardized rates of BD among women of reproductive age (15–49 years) across 204 countries and territories from 1990 to 2021.**(A)**. EAPC of ASIR;**(B)**. EAPC of ASPR;**(C)**. EAPC of ASDR.

**Figure 6:**
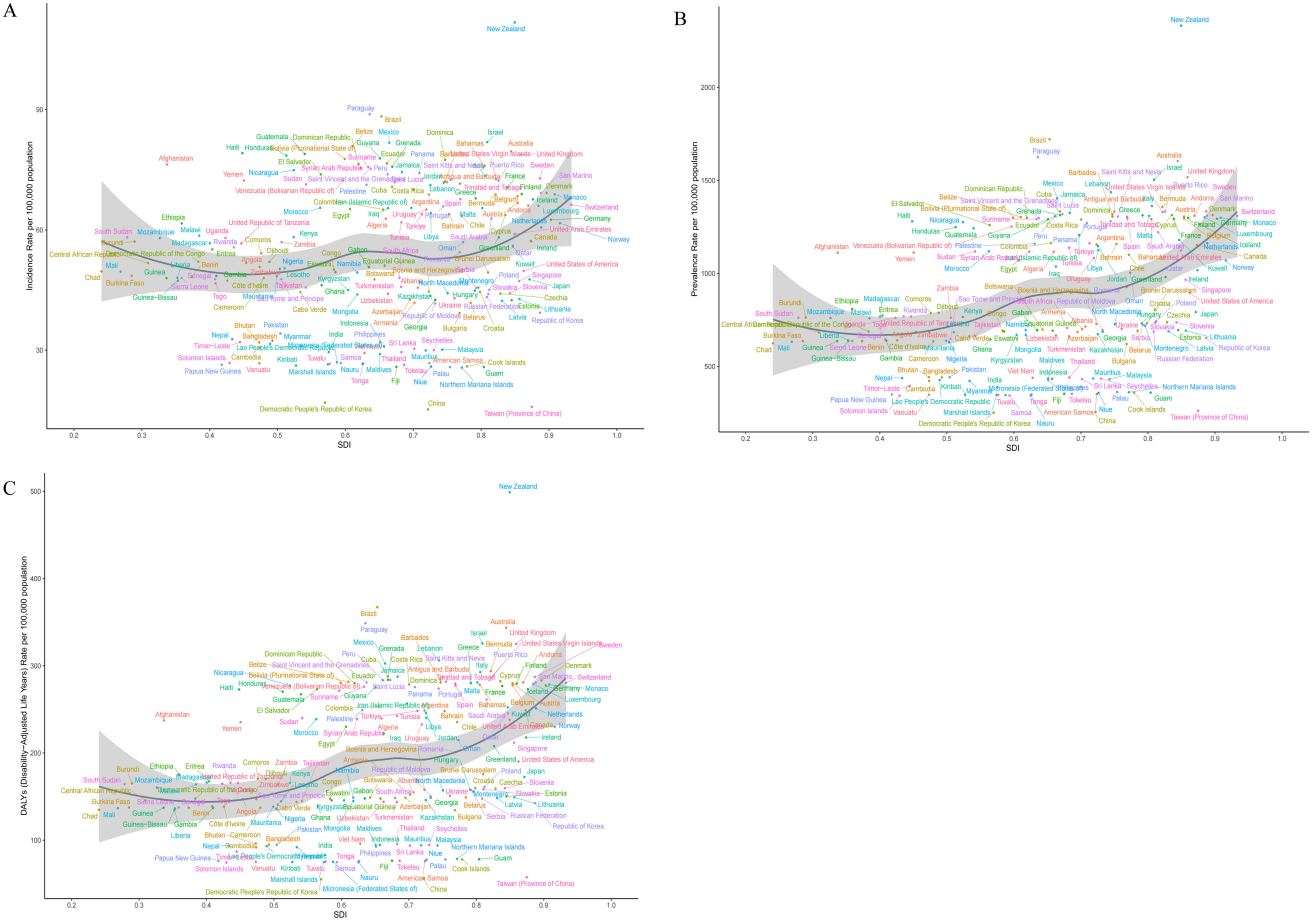
Associations between the SDI and the burden of BD among women of reproductive age across 204 countries and territories worldwide: **(A)**. Association between the age-standardized incidence rate of BD and SDI;**(B)**. Association between the age-standardized prevalence rate of BD and SDI;**(C)**. Association between the age-standardized DALYs rate of BD and SDI.

At the national level, in 2021, the ASIR of BD among women aged 15–49 varied widely across 204 countries, ranging from 15.23 to 111.75 per 100,000 population. New Zealand reported the highest ASIR at 111.75 per 100,000 (95% UI: 90.15–144.83), followed by Paraguay (88.88 per 100,000; 95% UI: 64.70–116.74) and Brazil (88.32 per 100,000; 95% UI: 71.54–110.59). In contrast, the lowest ASIR were observed in China (15.23 per 100,000; 95% UI: 12.17–19.06), Taiwan (15.85 per 100,000; 95% UI: 11.21–22.36), and the Democratic People’s Republic of Korea (16.94 per 100,000; 95% UI: 12.11–23.20) (**Supplementary Table 2**). The ASPR also showed substantial variation across countries, ranging from 251.99 to 2,331.74 per 100,000. New Zealand again ranked first with an ASPR of 2,331.74 per 100,000 (95% UI: 1,900.36–2,879.69), followed by Brazil (1,721.50 per 100,000; 95% UI: 1,438.36–2,068.58) and Paraguay (1,624.54 per 100,000; 95% UI: 1,151.92–2,264.03). The lowest ASPR were reported in the Democratic People’s Republic of Korea (251.99 per 100,000; 95% UI: 170.99–343.07), China (255.12 per 100,000; 95% UI: 206.75–307.72), and Taiwan (262.06 per 100,000; 95% UI: 176.21–353.71). ASDR ranged from 55.23 to 498.86 per 100,000 across countries. New Zealand had the highest ASDR at 498.86 per 100,000 (95% UI: 321.34–752.87), followed by Brazil (367.19 per 100,000; 95% UI: 241.55–538.51) and Paraguay (348.79 per 100,000; 95% UI: 207.40–529.84). The lowest ASDR were observed in the Democratic People’s Republic of Korea (55.23 per 100,000; 95% UI: 31.20–85.44), China (56.13 per 100,000; 95% UI: 35.95–82.81), and Taiwan (57.71 per 100,000; 95% UI: 32.73–86.04) (**Supplementary Table 2**). From 1990 to 2021, notable national-level trends were observed. Greenland had the largest increase in ASIR (EAPC = 0.42; 95% CI: 0.28–0.57), while Iran experienced the most pronounced decline (EAPC = –0.92; 95% CI: –1.06 to –0.78). Regarding ASPR, Iceland showed the greatest decrease (EAPC = –0.25; 95% CI: –0.32 to –0.19), whereas the Maldives had the highest increase (EAPC = 0.25; 95% CI: 0.22–0.29). Similar trends were observed in ASDR, with Iceland exhibiting the largest decline (EAPC = –0.25; 95% CI: –0.32 to –0.19), and the Maldives showing the steepest increase (EAPC = 0.25; 95% CI: 0.22–0.28) (**Supplementary Table 2**; **Figures 5A–C**). The relationship between SDI and disease burden from 1990 to 2021 showed a weak but nearly significant positive correlation between ASIR and SDI (Spearman’s r = 0.14; 95% CI: 0.02–0.25; p = 5.20×10⁻²), suggesting a slight upward trend in incidence in higher-SDI countries. In contrast, stronger and statistically significant positive correlations were observed between SDI and both ASPR and ASDR. Specifically, SDI was moderately positively correlated with ASPR (r = 0.42; 95% CI: 0.31–0.52; p = 5.04×10⁻^10^) and ASDR (r = 0.42; 95% CI: 0.31–0.51; p = 6.43×10⁻^10^) (**Figures 6A–C**), indicating that higher levels of socioeconomic development are associated with significantly increased prevalence and disability burden of BD among women of reproductive age.

### Cross-country inequality analysis

**Figure 7:**
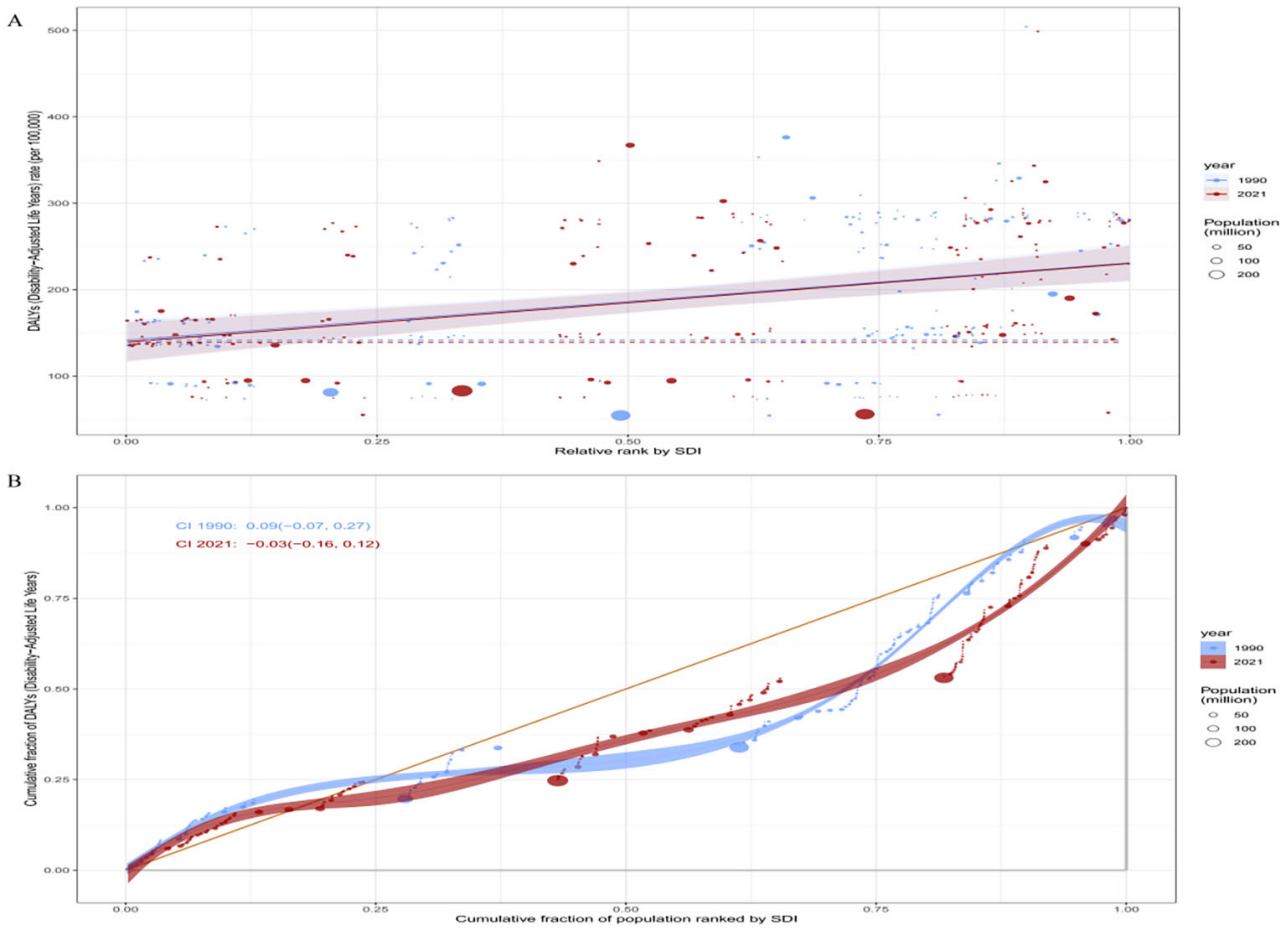
Health inequality analysis of the global burden of BD among women of reproductive age in 1990 and 2021. **(A)**: SII for ASDR due to BD among women of reproductive age in 1990 and 2021; **(B)**: CII for ASDR due to BD among women of reproductive age in 1990 and 2021.

Health inequality analysis of the ASDR for bipolar disorder among women of reproductive age (15–49 years) across 204 countries revealed a persistent imbalance in disease burden across countries with different levels of socioeconomic development. According to the SII, the ASDR gap between high-SDI and low-SDI countries was 89.57 per 100,000 (95% CI: 56.71–122.44) in 1990 and slightly increased to 90.97 per 100,000 (95% CI: 60.35–121.59) in 2021, suggesting that absolute inequality related to SDI has shown little improvement over the past three decades (**Figure 7A**). This finding indicates that despite global efforts to expand mental health service systems, the impact on reducing disparities in disease burden across socioeconomic strata remains limited. The concentration curve lay below the line of equality, indicating that the burden of bipolar disorder was primarily concentrated in countries with higher socioeconomic status (**Figure 7B**). However, the CII decreased from a positive value of 0.09 in 1990 (95% CI:–0.07 to 0.27) to a negative value of –0.03 in 2021 (95% CI:–0.16 to 0.12), suggesting a possible shift of the disease burden toward countries with lower SDI over time. This trend reflects a growing convergence in mental health challenges across nations with varying development levels, highlighting the urgent need for more targeted and equity-oriented mental health intervention strategies.

### Predicted trends

**Figure 8:**
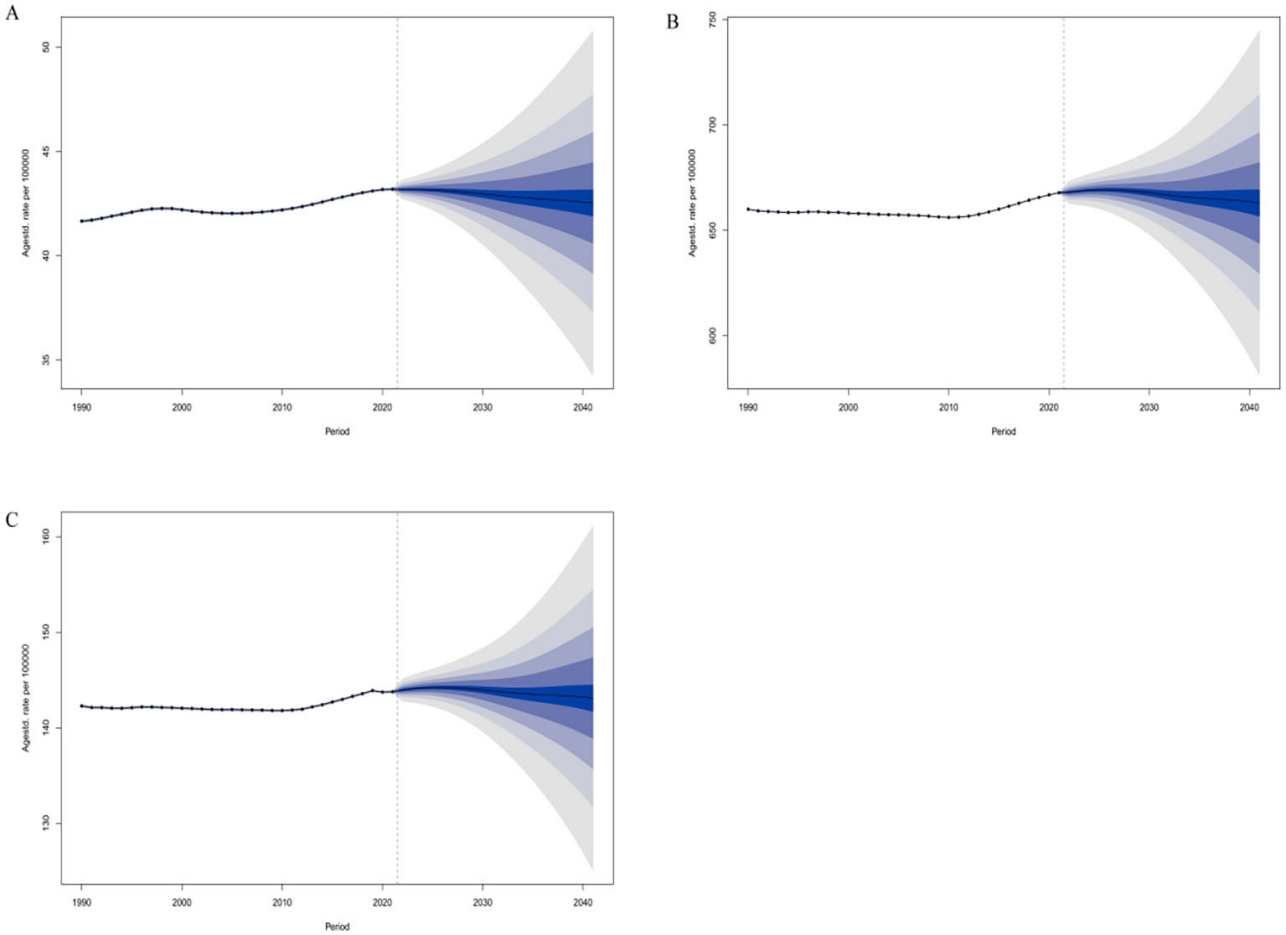
Predicted global changes in age-standardized rates of BD among women of reproductive age (15–49 years) by 2041:**(A)**. ASIR; **(B)** ASPR;**(C)**. ASDR.

We employed a BAPC model to project future trends in bipolar disorder among women of reproductive age (15–49 years) worldwide. The results indicated that by 2041, the global ASIR in this population is expected to decline slightly to 42.53 per 100,000 (95% UI: 32.70–52.37) compared to 2021 (**Figure 8A**). During the same period, the ASPR is projected to decrease to 663.00 per 100,000 (95% UI: 565.59–760.41), and the ASDR is expected to reach 143.11 per 100,000 (95% UI: 121.63–164.59), both showing a modest downward trend (**Figures 8B, C**). These forecasts suggest that although bipolar disorder will remain a significant public health concern for women of reproductive age in the coming decades, key standardized indicators may show some degree of alleviation. This indicates that recent advances in screening, diagnosis, and intervention in mental health may be gradually yielding positive outcomes. However, it is noteworthy that the prediction intervals remain relatively wide, highlighting the uncertainty of future trends. This underscores the need for continued attention to potential risk factors, particularly by enhancing the identification of high-risk populations and implementing sustained, integrated prevention and control strategies to consolidate the gains already achieved.

## Discussion

BD is a chronic, severe mental illness that typically begins in adolescence or early adulthood, characterized by high relapse rates, substantial disability, and a lifelong disease course. Its hallmark is the cyclical alternation between manic and depressive episodes, which severely impairs emotional stability, cognitive function, and the ability to fulfill social roles, ultimately reducing quality of life and increasing the public health burden (1, 20). Given the psychological and physiological vulnerability of women of reproductive age—due to the combined pressures of marriage, childbearing, career, and family—systematically evaluating the epidemiological characteristics of BD in this population is crucial for developing gender-sensitive and stage-specific mental health interventions. Our study shows that from 1990 to 2021, the number of incident cases, prevalent cases, and DALYs due to BD among women aged 15–49 years worldwide has steadily increased. Although the ASIR slightly declined, both the ASPR and ASDR continued to rise. This pattern—an overall increase in disease burden despite divergent trends in age-standardized rates—reflects a “burden shift” driven by population structural changes and enhanced disease recognition. On one hand, the global increase in the reproductive-age female population—particularly in low- and middle-SDI countries—has coincided with heightened susceptibility due to overlapping physiological changes (e.g., menstruation, pregnancy, postpartum hormonal shifts) (5, 21, 22), social pressures (e.g., career advancement, fertility anxiety, family responsibilities) (23, 24), and cultural constraints (e.g., low help-seeking behavior, mental illness stigma) (25–27). These factors contribute to increased BD vulnerability in this demographic, resulting in rising absolute case numbers and DALYs even when per capita risk remains relatively stable. On the other hand, BD is characterized by a high recurrence rate and the progressive accumulation of functional impairment. Many patients fail to receive timely diagnosis and intervention after the first episode, thereby entering a chronic and relapsing course that gradually increases the overall disease burden. Meanwhile, with the improvement of global mental health systems—particularly in high-SDI countries—the wider availability of diagnostic tools and heightened public awareness have substantially increased the detection and treatment rates of BD. Consequently, previously undiagnosed or misdiagnosed mild and subclinical cases have been incorporated into epidemiological records, contributing to the continued rise in ASPR and ASDR. However, it should be noted that data quality and regional variations in diagnostic practices may also affect the interpretation of these trends. In some low- and middle-SDI regions, especially in East Asia and sub-Saharan Africa, limited mental health resources, cultural stigma, and high rates of underdiagnosis may lead to an underestimation of the true disease burden. Therefore, the observed trends likely reflect the combined effects of genuine epidemiological changes, improvements in diagnostic coverage, and disparities in healthcare accessibility, rather than a simple increase in disease incidence. Overall, the rising burden of BD among women of reproductive age is the result of multiple intertwined factors, including population expansion, chronic disease progression, enhanced diagnostic rates, and improved data capture. These findings highlight the need for coordinated strategies to enhance diagnostic precision and strengthen mental health infrastructure, particularly in resource-limited settings.

Age-specific analysis in this study revealed that between 1990 and 2021, the number of incident cases, prevalent cases, and DALYs due to BD among women aged 15–49 years increased across all age groups globally, with marked heterogeneity in the rate of increase among different age brackets. Women aged 35–39 years exhibited the greatest rise in incident cases, suggesting that this stage represents a critical high-risk period for BD onset. This age group typically experiences overlapping pressures from career demands and family responsibilities, including childcare, eldercare, and workplace competition. Additionally, for some individuals, unresolved postpartum mood disturbances or progression from depressive disorders to bipolar spectrum disorders may further elevate the risk of disease onset. Women aged 45–49 years showed the most significant increase in both prevalence and DALYs, indicating a heightened vulnerability to disease exacerbation and functional decline during this period. Perimenopausal hormonal fluctuations, particularly declining estrogen levels, can substantially disrupt emotional regulation (28, 29). These physiological changes, combined with chronic comorbidities, shifts in family structure, and transformations in social roles, may accelerate the accumulation of disability. In terms of age-standardized rates, adolescent females aged 15–19 years had the highest ASIR in 2021, reflecting their increased sensitivity to novel psychosocial stressors—such as social media exposure, online environments, and academic pressure—during a developmental stage characterized by immature emotional regulation and heightened neuropsychological plasticity (30). Meanwhile, women aged 25–29 years had the highest ASDR and ASPR, indicating this stage as a peak period for both disease prevalence and functional impairment. This age range often coincides with transitions in marriage, childbirth, employment, and social identity, creating substantial psychological demand that is often unmet by existing mental health service coverage. Therefore, targeted, age-specific interventions are urgently needed. Emotional literacy and resilience training should be prioritized for adolescent girls, while reproductive and workplace support systems must be strengthened for young women. For middle-aged women, greater emphasis should be placed on managing perimenopausal mental health. Crucially, integrated and continuous mental health support systems should be established in key settings such as schools, communities, and workplaces to improve early screening and treatment rates, preserve functional capacity, and ultimately reduce the long-term burden of BD in this population.

From the perspective of SDI stratification, the period from 1990 to 2021 saw particularly pronounced increases in the absolute burden of BD among women of reproductive age—measured by incident cases, prevalent cases, and DALYs—in low and lower-middle SDI regions. This trend reflects a cumulative risk release driven by the dual forces of demographic expansion and unequal distribution of mental health resources. Fueled by population momentum, the number of women of reproductive age has surged in these regions. Coupled with gradual advances in mental health awareness campaigns and basic screening initiatives, previously “silent” disease burdens have begun to surface, forming a feedback mechanism of “improved recognition leading to burden visibility.” Although this shift signals a growing awareness of mental health issues, the post-diagnosis treatment and rehabilitation systems have largely failed to keep pace. In contexts characterized by fragmented chronic disease management, extreme shortages of psychiatric specialists, and limited coverage by primary care systems, many diagnosed individuals remain “identifiable but untreatable.” Consequently, both ASPR and ASDR have continued to rise, contributing to worsening functional impairment and disability-related consequences (31). In contrast, high-SDI regions, despite having robust health infrastructures and relatively well-developed mental health systems, still recorded the highest ASIR, ASPR, and ASDR globally in 2021. This “high recognition–high burden” paradox does not necessarily indicate a greater disease prevalence but likely reflects heightened diagnostic sensitivity, broader screening coverage, and the ongoing destigmatization of mood disorders. In high-income countries, women of reproductive age often juggle multiple roles—including career progression, childbearing, and caregiving—making them more vulnerable to emotional stress and subclinical mental distress in high-pressure environments. Furthermore, the high utilization of healthcare services, widespread mental health education, and systematic diagnostic criteria contribute to more comprehensive identification and reporting of BD cases (1, 32). Importantly, elevated ASPR and ASDR in high-SDI settings should not be interpreted solely as indicators of policy failure. Rather, they may reflect a “stockpile effect” of chronic illness—where improved survival and prolonged disease duration lead to cumulative burden over time. This aligns with a transition into the chronic management phase of BD, indicating initial success in early detection and acute-phase treatment. Overall, the global SDI-related distribution of BD burden underscores a mismatch between mental health resource allocation and disease burden patterns. High-income countries face the challenge of chronic burden accumulation amid high recognition rates, whereas low- and middle-income countries struggle with rapidly escalating burdens and delayed interventions. Addressing this asymmetry requires future global mental health strategies to simultaneously enhance health equity and system resilience.

At the regional level, the burden of BD among women of reproductive age (15–49 years) demonstrates pronounced global geographic disparities. In 2021, South Asia ranked first worldwide in terms of incident cases, prevalent cases, and DALYs. This is not only attributable to its large population base but also reflects a structural contradiction between accelerating urbanization and lagging mental health service provision. Although legislative reforms and foundational mental health service infrastructure have made progress in some countries, low recognition rates, delayed diagnoses, and uneven resource distribution remain pervasive. Women in this region are often subjected to compounded stressors including domestic responsibilities, labor demands, and cultural oppression. Persistent gender inequality and the lack of psychological support further heighten the risk of mood disorders (33). In contrast, the burden of BD in Oceania was the lowest globally in 2021, which may be explained by its relatively small population and well-established community-based mental health support systems, along with a high level of public awareness regarding mental health. East Asia reported the lowest ASIR, ASPR, and ASDR values globally. This phenomenon may partly result from the stigma surrounding mental illness and cultural differences in symptom expression, leading to underdiagnosis and underreporting. However, recent efforts in countries like China and Japan to build multi-tiered mental health service systems—especially at the university and community levels—have facilitated early intervention and may have curbed disease progression to some extent. In Tropical Latin America, BD burden was amplified by a more developed psychiatric service and surveillance system, contributing to higher case identification and reporting rates. Additionally, the prevalent use of substances such as alcohol and cocaine has further triggered or exacerbated manic and depressive episodes, making this region the global leader in ASIR (34, 35). On the other hand, Australasia had the highest ASPR and ASDR globally, indicating that although incidence rates are not particularly high, disease course tends to be prolonged, with frequent relapses and severe functional impairments. This suggests that while acute-phase intervention may be adequate, long-term rehabilitation and social support systems remain underdeveloped. Trend analyses reveal that ASIR is declining in most regions; however, exceptions such as high-income North America and Western Europe show a continued upward trend. This could be attributed to increased diagnostic sensitivity, wider screening coverage, and better healthcare accessibility. It may also reflect the “diagnostic expansion effect” due to broader application of standardized criteria such as DSM. Conversely, regions like Southeast Asia have experienced marked increases in ASPR and ASDR, indicating that although recognition capacity is improving, effective treatment and rehabilitation services are still lacking, leading to a shift toward chronicity in BD. Further correlation analyses between SDI and disease burden reveal a slight positive correlation between ASIR and SDI, and moderate positive correlations between ASPR/ASDR and SDI. This suggests that as socioeconomic development advances, disease recognition and reporting improve, but so do survival rates and the identification of functional impairments—producing what may be termed a “representation effect.” In other words, although high-SDI regions benefit from improved diagnosis and care, they also accumulate larger cohorts of chronic psychiatric patients, manifesting a “mental health paradox”—where medical progress enhances survival and quality of life, yet simultaneously magnifies and prolongs the functional and social burden of mental disorders.

At the national level, the burden of BD among women of reproductive age (15–49 years) across 204 countries and territories displays pronounced geographic disparities. In 2021, countries such as New Zealand, Brazil, and Paraguay ranked among the highest globally in terms of ASIR, ASPR, and ASDR. This may be attributed to well-established mental health systems, standardized diagnostic protocols, and heightened public awareness of mental health in these countries. The widespread adoption of the DSM diagnostic framework (36, 37) has likely enhanced the detection of BD spectrum disorders, although it may also contribute to a degree of “diagnostic expansion.” Furthermore, persistent social stress and high rates of violence in some Latin American countries may create an environment of heightened vulnerability to affective disorders. In contrast, countries such as China, Taiwan, and Democratic People’s Republic of Korea recorded some of the lowest ASIR, ASPR, and ASDR levels worldwide. This could be linked to persistent stigma surrounding mental illness (38), internalized symptom expression among women (39), low rates of healthcare utilization, and limited mental health service coverage. Additionally, milder forms of BD may often be misdiagnosed as depressive disorders in primary care settings, leading to systemic underestimation of the true burden. Trend analyses indicate that the BD burden is evolving dynamically in several countries. For example, Greenland has experienced a sustained increase in ASIR, likely reflecting improvements in case detection. In contrast, countries like Iran and Iceland have shown declines in ASIR or ASPR, potentially due to policy advances such as earlier intervention, improved adherence management, and relapse prevention strategies. The rising ASPR and ASDR in the Maldives may reflect insufficient psychological adaptation mechanisms and delayed responses to mental health risks during periods of sociocultural transition. With regard to the relationship between the SDI and BD burden, this study found a mild positive correlation between ASIR and SDI, suggesting that high-SDI countries have more complete identification of BD without necessarily experiencing a substantial increase in incidence. In contrast, ASPR and ASDR were moderately positively correlated with SDI, indicating a larger “disease stockpile” and accumulated functional burden in higher-SDI settings. On one hand, well-developed healthcare systems prolong patient survival, thereby enabling more comprehensive documentation of chronic disease trajectories. On the other hand, psychosocial stressors—such as fast-paced lifestyles, increased role conflict, and rising loneliness—may contribute to elevated risk, particularly among women of reproductive age. Importantly, substantial variations in BD burden are observed even among countries with comparable SDI levels. This underscores the role of additional structural factors—such as healthcare infrastructure, cultural perceptions, service delivery models, family support systems, and gender equality policies—in shaping disease risk and disability outcomes. Therefore, national BD prevention and control strategies must be context-specific, informed by sociocultural dynamics and healthcare capacity, and structured to support targeted, stratified, and sustainable interventions.

This study’s health inequality analysis reveals that the burden of BD among women of reproductive age (15–49 years) has remained markedly unequal across countries with varying levels of socioeconomic development. From 1990 to 2021, the ASDR of BD consistently exhibited a gap of nearly 90 per 100,000 population between high- and low-SDI countries, with no significant change in the SII. This persistent disparity suggests that, despite recent efforts to reform mental health service systems worldwide, progress toward equitable global mental healthcare remains sluggish—constrained by differences in financial investment, availability of mental health professionals, infrastructure, and service accessibility. Spatially, the concentration curve for 1990 lay below the equality line, indicating that the BD burden was predominantly concentrated in high-SDI countries. This may be explained by higher rates of diagnosis, reduced stigma, and more comprehensive mental health systems in those settings. However, the CII shifted from a positive value in 1990 to a near-zero or slightly negative value by 2021, reflecting a gradual redistribution of the BD burden toward low- and middle-SDI countries. This trend may be driven by accelerating social transformation, increased urban stressors, and persistent gender inequalities in those regions, as well as improved awareness and detection of mental health conditions. Importantly, while high-SDI countries continue to bear the largest absolute burden of BD, their rate of increase has plateaued. In contrast, low- and middle-SDI countries are experiencing a rapid rise in BD burden, facing the compounded challenges of shifting disease epicenters, evolving burden structures, and insufficient response capacity. These findings underscore the urgent need for the international community to monitor and respond to the evolving landscape of mental health inequality. Specifically, there is a pressing need to establish “capacity-compensatory” mental health support mechanisms targeted at low- and middle-SDI countries, prioritize high-risk nations and populations, and advance a more precise and equitable model of global mental health governance.

Predictive analysis indicates that although the ASIR, ASPR, and ASDR of BD among women aged 15–49 are projected to show a modest decline over the coming decades, the overall disease burden will remain relatively high, underscoring BD as a long-term global public health challenge for women’s health. This trend may be partly attributable to the phased progress achieved in recent years across many countries, particularly in high-SDI regions, in areas such as mental health policies, screening systems, clinical guidelines, and public awareness. For instance, the establishment of systematic early screening mechanisms, the optimized allocation of psychiatric resources, and the widespread use of mood-stabilizing treatments may have contributed to curbing new cases and mitigating adverse disease progression. At the same time, it should not be overlooked that another key driver of the projected decline lies in the transformation of the global population structure, especially the reduction in the size of younger age groups, which is particularly pronounced in the context of accelerated population aging and declining fertility rates in certain regions. Nevertheless, the wide prediction intervals highlight that considerable uncertainty remains regarding the future burden. The trajectory of mental disorders is shaped by numerous interacting variables—such as social transitions, environmental crises, economic fluctuations, digital-era stressors, and the stability of social support systems—the nonlinear interplay of which may lead to fluctuations in disease burden and even “rebound” risks in some regions (40, 41). Additionally, unforeseen events such as post-pandemic mental health crises, global conflicts, and climate emergencies could further heighten psychological vulnerability in women of reproductive age, undermining disease control efforts. To address this, global mental health governance must evolve from treatment-focused models toward a full-cycle framework encompassing prevention, early detection, intervention, and rehabilitation. Special attention should be given to low- and middle-SDI countries and marginalized female populations, with efforts to improve accessibility, adaptability, and cultural sensitivity of mental health services. Key strategies include: (1) Strengthening integrated mental health care within primary health systems should include capacity-compensatory mechanisms, such as standardized training for primary care providers, task-shifting to community health workers, remote specialist support, provision of essential psychotropic medications, and mobile clinics or outreach programs to address service gaps in resource-limited areas; (2) early screening should be incorporated into existing school health programs and maternal-child health services, using validated brief assessment tools during routine visits or immunization schedules to identify high-risk individuals without imposing substantial additional resource demands; (3) mental health literacy and empowerment initiatives delivered through community activities, peer-support networks, and culturally tailored digital platforms can reduce stigma and encourage timely help-seeking; and (4) data-driven risk prediction and regional surveillance using electronic health records and centralized registries can monitor trends, detect emerging high-risk populations, and guide targeted interventions. Only through the establishment of multi-level, cross-sectoral collaborative mechanisms can the long-term rise in bipolar disorder burden among women be effectively curbed, achieving equitable global mental health outcomes.

This study has several key strengths. Relying on the most recent data from the GBD 2021, it systematically analyzed trends and spatial heterogeneity in the burden of BD among women aged 15–49 years across 204 countries and territories from 1990 to 2021. The study spans a 32-year time series, offering strong global representativeness and valuable cross-regional comparability. In the context of rising mental health risks among women, these findings provide an important evidence base for developing gender-sensitive and regionally tailored mental health policies. However, several limitations should be noted. First, the identification, diagnosis, and reporting of BD vary across countries and cultural contexts. In low-SDI countries, limited health resources, social stigma, and underdeveloped data collection systems may result in incomplete case reporting, necessitating model-based estimations. Such data scarcity could introduce systematic bias, potentially underestimating the disease burden in low-SDI regions and affecting the interpretation of global and regional burden trends. Second, although the GBD model integrates multiple data sources to enhance estimation robustness, its results remain constrained by the quality of the original data. Psychiatric disorders heavily rely on subjective assessments, and cross-cultural comparability is challenging, which may lead to systematic differences in burden estimates across regions. Third, this study focuses on women of reproductive age but does not further stratify BD subtypes (e.g., type I vs. type II), comorbidities such as suicide risk, or perinatal mood disorders, limiting the precise identification of high-risk subgroups and potentially affecting the formulation of targeted intervention strategies. Fourth, although we employed the BAPC model to project future disease burden, this approach is based on historical trends and cannot dynamically account for potential interventions, policy reforms, social stressors, or technological advances, resulting in inherent uncertainty in the projections. Finally, this study did not systematically incorporate social and cultural determinants (e.g., marital status, educational attainment, social support networks), which may introduce residual confounding when interpreting differences in burden across countries and regions.

## Conclusions

From 1990 to 2021, the ASIR of BD among women of reproductive age showed a slight decline globally, whereas the ASPR and ASDR continued to rise. This trend highlights multiple challenges, including disease chronicity, accumulation of functional impairment, and inadequate long-term management. The burden of BD demonstrates marked heterogeneity across age, geographic regions, and socio-demographic strata. Although high-SDI countries exhibit a relatively higher burden, the trends have largely stabilized. In contrast, low- and middle-SDI countries have experienced a sustained increase in burden driven by population growth and improved detection rates. Health inequality analysis indicates that over the past three decades, disparities in BD burden across SDI levels have not substantially narrowed, with the burden increasingly shifting toward low- and middle-income countries. Forecasts suggest that while ASIR, ASPR, and ASDR may slightly decline over the next two decades, the overall burden will likely remain high, underscoring the continued severity of the global mental health challenge. Given their elevated vulnerability, women of reproductive age should be prioritized as a key target population in global mental health policies. There is an urgent need to develop more regionally tailored intervention strategies, strengthen early screening and sustained treatment, enhance service accessibility and continuity, and bolster international collaboration and resource allocation. Such efforts are essential to effectively reduce the BD-related burden in women of reproductive age and to improve women’s mental health globally.

## Data Availability

The original contributions presented in the study are included in the article/**Supplementary Material**. Further inquiries can be directed to the corresponding authors.
